# Neural set point for the control of arterial pressure: role of the nucleus tractus solitarius

**DOI:** 10.1186/1475-925X-9-4

**Published:** 2010-01-11

**Authors:** B Silvano Zanutto, Max E Valentinuzzi, Enrique T Segura

**Affiliations:** 1 Instituto de Ingeniería Biomédica (IIBM), Facultad de Ingeniería (FI) Universidad de Buenos Aires (UBA), Av Paseo Colón 850, C1063ACV, Buenos Aires, Argentina; 2 Instituto de Biología y Medicina Experimental (IBYME)-CONICET, Vuelta de Obligado 2490, C1428ADN - Buenos Aires, Argentina

## Abstract

**Background:**

Physiological experiments have shown that the mean arterial blood pressure (MAP) can not be regulated after chemo and cardiopulmonary receptor denervation. Neuro-physiological information suggests that the nucleus tractus solitarius (NTS) is the only structure that receives information from its rostral neural nuclei and from the cardiovascular receptors and projects to nuclei that regulate the circulatory variables.

**Methods:**

From a control theory perspective, to answer if the cardiovascular regulation has a set point, we should find out whether in the cardiovascular control there is something equivalent to a comparator evaluating the error signal (between the rostral projections to the NTS and the feedback inputs). The NTS would function as a comparator if: a) its lesion suppresses cardiovascular regulation; b) the negative feedback loop still responds normally to perturbations (such as mechanical or electrical) after cutting the rostral afferent fibers to the NTS; c) perturbation of rostral neural structures (RNS) to the NTS modifies the set point without changing the dynamics of the elicited response; and d) cardiovascular responses to perturbations on neural structures within the negative feedback loop compensate for much faster than perturbations on the NTS rostral structures.

**Results:**

From the control theory framework, experimental evidence found currently in the literature plus experimental results from our group was put together showing that the above-mentioned conditions (to show that the NTS functions as a comparator) are satisfied.

**Conclusions:**

Physiological experiments suggest that long-term blood pressure is regulated by the nervous system. The NTS functions as a comparator (evaluating the error signal) between its RNS and the cardiovascular receptor afferents and projects to nuclei that regulate the circulatory variables. The mean arterial pressure (MAP) is regulated by the feedback of chemo and cardiopulmonary receptors and the baroreflex would stabilize the short term pressure value to the prevailing carotid MAP. The discharge rates of rostral neural projections to the NTS would function as the set point of the closed and open loops of cardiovascular control. No doubt, then, the RNS play a functional role not only under steady-state conditions, but also in different behaviors and pathologies.

## Introduction

Cardiovascular variables are regulated by humoral, neural and autoregulatory mechanisms. It is generally accepted that the renal output curve takes care of long-term arterial blood pressure, but the possible role of the nervous system in it has not been fully understood yet. Guyton, in 1991 [1], noted that *many prominent researchers believe that much, if not most hypertension in human beings is initiated by nervous stress. But how can stress cause hypertension*? Measurements of arterial pressure show large variations over a 24-hour period and often leave diagnostic doubts, reported Drayer *et al*, in 1985, and Weber, in 1988 [2,3]. For example, acute emotional or threatening stimuli can also elicit a marked cardiovascular response (as in the classic "defense" or "alert" response). Electrical stimulation of a region in the hypothalamus, referred to as the "defense area", elicits a cardiovascular response very similar to that described above [4,5]. Besides, neurally-mediated cardiovascular responses are also evoked as part of other more complex behaviors; for example, the onset of exercise is followed and in some case preceded by an immediate increase in arterial pressure (about 15-20%,), heart rate and ventilation [6]. The latter are accompanied by an increase in skeletal muscle blood flow and rise in the activity of sympathetic nerves to other vascular beds, such as the kidneys [7]. The cardiovascular and respiratory changes that occur at the onset of exercise have been shown to be a consequence of "central command", initiated from the cortex at the same time as the somatomotor activity increases [8]. In other words, the level around which arterial pressure fluctuates or is regulated, that is, the ***set point ***(or a reference value), varies under different conditions. Not long ago in a review paper, Osborn *et al*, in 2005 [9] proposed that a "baroreflex independent" sympathetic control system must exist for the long-term regulation of sympathetic nerve activity and arterial pressure, discussing also the concept of a central nervous system "set point" and its involvement in the pathogenesis of hypertension. Besides, Montani and Van Vliet [10] made a quick summary as introduction to a series of articles on the subject, which, as they state, is still not fully settled. The history of the baroreceptors has been told by Persson, in 1991 [11].

The objective herein is to collect information from the literature at large and from our own experience that, in our view, is close enough to ascertain that **there is a neural set point for the long term control of blood pressure**. This paper reviews the subject, too.

## Antecedents

It is well known that in the neural control circuit of the circulatory system some cardiovascular variables are fedback by arterial and cardiopulmonary receptors. Arterial receptors are of two types, baroreceptors, that is, stretch structures located in the walls of the carotid sinuses and the aortic arch, and chemoreceptors located in the carotid and aortic bodies. Today, it is generally accepted that short-term blood pressure (i.e., seconds to minutes) is regulated by a negative feedback loop and that the information from the baroreceptors is very effective to stabilizing such changes (say, for example, during orthostatism). Thus, these receptors are the major sense organs which reflexly control systemic arterial blood pressure. Historically, the French physiologist Marey, in 1859, was the first to recognize the inverse relationship between arterial pressure and heartrate (known as Marey's law). Around 1861, J. B. A. Chauveau developed a procedure (Chauveau-Marey maneuvre) to manually introduce a sudden blood pressure step to trigger the reflex compensatory heart rate response [12,13].

Thus, any change in pressure modifies baroreceptor discharge and, through modifications in the autonomic output, blood pressure returns to the basal value. A baroreceptor function curve offers a good description, which usually displays a sigmoid appearance. During hypertension, for example, this curve is displaced toward a higher value and the operating point resets to the prevailing carotid pressure, facts well documented by McCubbin and collaborators in 1956 and, later on, by Kunze, in 1981 [14-16]. This means that the same pressure value can be associated with different discharge patterns depending on the long-term blood pressure level, which implies that there is no definite relationship between a value of mean arterial blood pressure and baroreceptor activity, suggesting that this mechanism is not involved in the long-term regulation.

**Figure 1:**
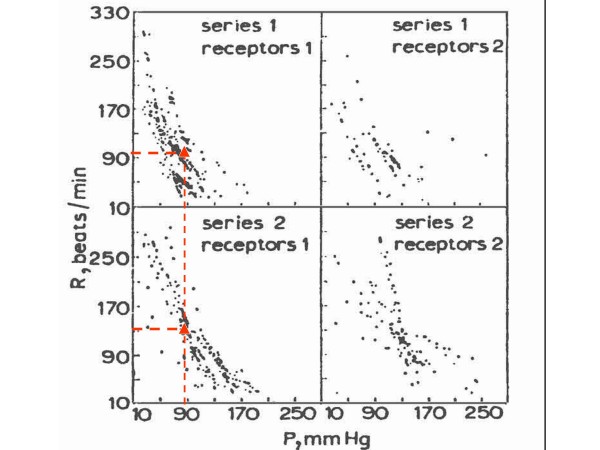
**Experimental baroreceptor function**. Curves in four different conditions where the controlling variable was cardiac frequency increased or decreased by parasympathetic (vagus nerves) discharge, in turn driven from the medulla oblongata outflow. If the horizontal scale is turned over, the sigmoid shape becomes more evident. Triangles on the two left panels mark probable operating points. (Modified after Valentinuzzi, Powell et al, 1972, ref [13], by permission).

**Figure 2:**
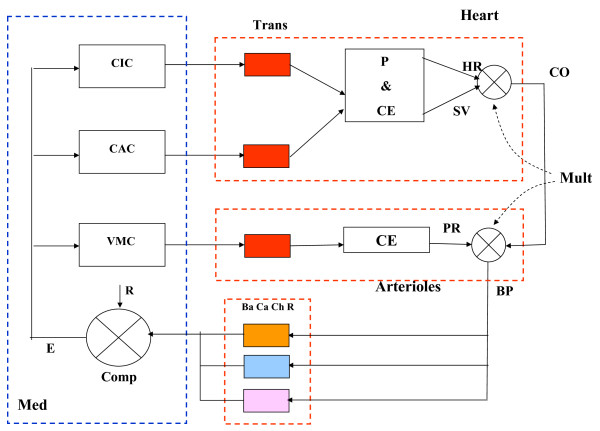
**Baro-Cardiopulmonary-Chemoreceptors negative feedback loop**. Med, medulla oblongata; CIC, CAC, VMC, cardioinhibitory, cardioaccelerator and vasomotor centers, respectively; Comp, comparator; neural reference R; error signal E after the difference against the outflow from the baro, cardiopulmonary and chemoreceptors (Ba, Ca ChR), respectively. BP, blood pressure = PRxCO; Mult, multipliers; PR, peripheral resistance; CO, cardiac output = HRxSV; HR, heart rate; SV, stroke volume. P, pacemaker; CE, myocardial contractile fibers; Trans, postulated transducers from neural section to CV side. (Modified after Valentinuzzi, Powell et al, 1972, ref [13], by permission).

Figure 1 shows four real function curves, actually scattergrams, under different experimental conditions, in dogs, where the controlling variable was cardiac frequency, which changed by parasympathetic discharge, in turn driven from the medulla oblongata outflow, as described by Valentinuzzi *et al *[12,13]. The red triangles in the two left panels mark the approximate operating points for the same blood pressure (90 mmHg), but heart rate for the lower curve is much higher than in the upper curve, indicating a definite shift to the right (if the horizontal axis is turned over). These curves well illustrate the inverse relationship between heart rate and pressure. The series numbers refer to the type of anesthesia and receptors refer either to all baroreceptors or just only one portion. These papers [12,13] studied and proposed what was called control parameters of the arterial blood pressure system, defining heart rate sensitivity (expressed in beats/min change per unit of blood pressure change), which may be taken as baroreceptor sensitivity, and even trying to obtain a set point (in pressure units), the open-loop gain and the basal rate values. The set point value (might be also called "reference", although not everybody would agree) appeared as very close to the arterial mean pressure, besides, baroreflex sensitivity was significantly influenced by anesthesia. A block diagram in that paper suggests a reference pressure in terms of central nervous system output that is reproduced and modified herein as Figure 2. The medulla oblongata contains the cardioinhibitory, cardioaccelerator and vasomotor centers (CIC, CAC, VMC). We postulate also the existence of a comparator (Comp) with a neural reference R and its error signal after the difference against the outflow from the baro, cardiopulmonary and chemoreceptors (Ba, Ca, Ch), respectively. Actual blood pressure (BP) is derived from a postulated multiplication between cardiac output (CO) and peripheral resistance (PR), the latter as a result of the arterioles contractile elements. In turn, CO is supplied directly by the heart, after the product of heart rate (HR) and stroke volume (SV). The three cardiovascular centers act upon the three postulated transducers (Trans), which drive the pacemaker and the myocardial contractile fibers, thus completing the loop.

Furthermore, central nervous mechanisms modify baroreceptor sensitivity and, thereby, mediate the resetting of baroreceptors [15,17,18]. In all studies in which these receptors were denervated, blood pressure oscillations increased significantly while mean arterial pressure (MAP) did it only for a few days [19-26]. Even when both carotid and aortic baroreceptors and chemoreceptors were removed, MAP increased only for a short period of time [27]. One possible conclusion was that sensory-neural transduction is not involved in the long-term regulation of blood pressure.

Rather early in the endeavor, Granger and Guyton [28], in 1969, advanced that the stability of central physiological variables is achieved by "whole body" autoregulation through natriuresis and diuresis, while the failure of sinoaortic denervation to alter long-term level of arterial pressure was originally used as an argument against a role for the entire nervous system in arterial pressure regulation; such position was later on reaffirmed [29]. One reason is because over the past few decades the primary focus of many studies has been placed on neural control of the kidneys, considering this vascular bed as playing a major role in long-term control of arterial pressure. Increases in the renal autonomic neural system (ANS) function results in several responses that potentially and chronically elevate arterial pressure; they include sodium and water retention, increased activity of the renin-angiotensin-aldosterone system and increased renal vascular resistance [30]. Consistent with the water retention general idea, Cox, in 1989 [31], put forward, although perhaps not for the first time, the *water logging *concept in the arterial walls as a mechanism to permanently change their structure and, through it, peripheral resistance and, thus, blood pressure, while Guyton and colleagues proposed that the only mechanism by which the sympathetic nervous system can chronically regulate arterial pressure is via alterations in the renal function curve [32]. At this point, it must be underlined the highly significant contribution of Guyton, Jones and Coleman, as early as 1963 [33], with their classical and still valid book.

Quoting almost verbatim from Kunze [16], his experimental results indicated that after the pressure of an isolated perfused carotid sinus was held at 80 mmHg for 20 min, the threshold pressure necessary to elicit the reflex systemic blood pressure response was about 78 mmHg. When carotid pressure was maintained for 20 min at 120 and 160 mmHg, instead, the threshold rose to 113 and 126 mmHg, respectively. Such resetting of the threshold to a stable value upon elevating or reducing carotid sinus pressure was accomplished within 15-20 min. The entire range of operation of the reflex response was shifted to higher carotid pressures as the holding or clamped pressure was elevated while the midrange gain of the response was unchanged at the three holding pressures tested. These findings indicate that the carotid reflex need not operate over a fixed range but that the range may be rapidly adjustable to the prevailing pressure. When arterial pressure is sustained at a level that is elevated or depressed from normal, the carotid baroreceptor reflex acutely resets to operate in the range of the prevailing pressure with a threshold that has moved toward that pressure.

Guyton [34], in 1977, proposed that blood volume regulation takes place through the diuresis/natriuresis functions of the kidneys; the latter obviously coexisting with neural mechanisms controlling blood pressure. DiBona [30] noted that *alterations in efferent renal sympathetic nerve activity produce significant changes in renal blood flow, glomerular filtration rate, reabsorption of water, sodium, and other ions, and the release of renin, prostaglandins, and other vasoactive substances*. Moreover, chronic recordings in freely moving cats showed the presence of continuous background activity in the renal nerves [35]. This tonic efferent discharge is reduced by elevated blood pressure, increased by exercise, and almost eliminated by ganglionic blockade. Also, the loss of neurogenic vasomotor tone can reduce mean pressure from 100 mmHg to 50 mmHg or less, and injection of very small doses of norepinephrine can immediately restore the previous pressure [36]. Finally, renal denervation (under the tonic control of sympathetic premotor neurons in the RVLM has no effect on arterial pressure in normotensive animals [37].

Denervation of baroreceptors and chemoreceptors does not open the negative feedback loop, because there is another kind of receptors, the cardiopulmonary ones. These are mechano or stretch receptors located in the heart chambers and in lungs [38,39]. Cardiopulmonary afferent pathways seem to especially influence those neuron pools supplying the renal resistance vessels, whereas their action on those fibers to skeletal muscle resistance vessels is less pronounced. These receptors cannot sense rapid fluctuations in arterial pressure as arterial baroreceptors do. The cardiopulmonary afferent fibers were chronically denervated by dissecting all branches leading to the thoracic dog's vago-sympathetic trunk, as reported by Persson *et al*, in 1988 [27]. The acute cardiovascular denervation by cold block or acute dissection of these receptors would increase arterial pressure for only a short period of time, as seen after arterial baro and chemo receptor denervation [25,40]. Only by cardiopulmonary and arterial receptor denervation would the negative feedback be open. After combined denervation, sustained hypertension was found, as well as a large fluctuation in arterial pressure that characterizes arterial receptor denervation [41].

Thus, cardiopulmonary receptors are an irreplaceable component for determining mean arterial pressure in cardiovascular regulation. The feedback from cardiopulmonary and arterial receptors is not only confined to neural cardiovascular responses, but it is also involved in mechanisms for the release of renin, antidiuretic hormone, catecholamines and vasopressin [42]. These hormones might contribute to the above mentioned sustained hypertension. In this way, the CNS regulates the long-term blood pressure by the feedback of chemoreceptors and cardiopulmonary receptors. Based on these data, the question remains as whether there exists a set point of the cardiovascular control loop to regulate MAP. To answer it, first we should find out whether in the cardiovascular control there is something equivalent to a comparator evaluating the error signal (between the rostral nervous projections to the NTS and the feedback inputs).

### Analysis of the neural paths and role of the nucleus tractus solitarius (NTS) and its rostral structures

To study whether there is a neural structure functioning as comparator, the main paths involved in the cardiovascular regulation must be analyzed. The NTS receives fibers from baro- and chemoreceptors, mainly through the aortic depressor and the carotid sinus nerves [5,43-47]. As baroreceptor primary afferent fibers, chemoreceptor fibers terminate in the NTS [48,49]. The cardiopulmonary fibers converge to the same pool of central neurons as the arterial receptors and act in a similar way [50,51]. The NTS projects to neurons within the caudal and intermediate parts of the ventrolateral medulla (VLM) and it also projects to several brainstem nuclei: the lateral reticular nucleus and the nucleus gigantocellularis, among others [47,52]. Besides, there are links to a "depressor area" in the caudal ventrolateral medulla (CVLM), where inhibition of sympathetic excitatory neurons of a "pressor area" in the rostral ventrolateral medulla (RVLM) may take place [51,53,54]. Neurophysiological studies indicate that the major source of peripheral chemoreceptor drive to RVLM pre-sympathetic neurons is likely to originate from neurons located in the NTS [48,49]. Moreover, the nucleus sends fibers to the intermediolateral spinal column (IML) [55,56]. Efferents from the IML pre-sympathetic neurons innervate the myocardium and smooth muscle vessels and the NTS projects to the dorsal vagal nucleus and the nucleus ambiguous, which, in turn, send fibers to the heart [5].

The NTS as well as other key medullary nuclei subserving the baroreceptor reflex receive inputs from higher centers of the brain, including the hypothalamus and other forebrain regions with important roles in mediating cardiovascular responses to acute stresses. The hypothalamus sends fibers to the dorsal vagal nucleus, to the nucleus ambiguous, to the NTS, and to the intermediolateral cell column of the spinal cord [57,58]. It receives projections, too, from the amygdala through the stria terminalis and from the septum through the medial forebrain bundle [56,59,60]. Besides, the dorsomedial hypothalamic nucleus (DMH) projects directly to the NTS and a high proportion of these cells have collateral links to the RVLM [61].

**Figure 3:**
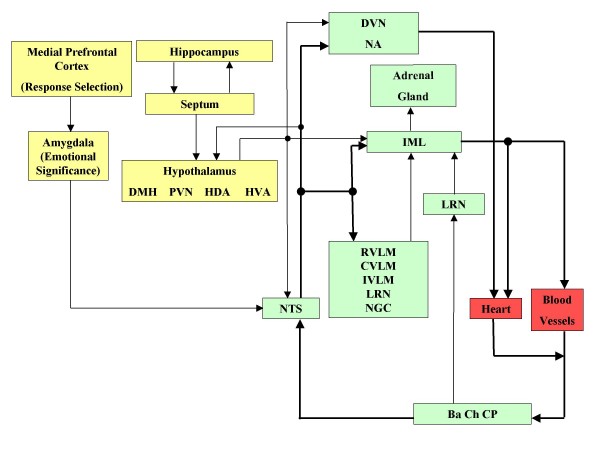
**Cardiovascular System Nervous Control**. The NTS receives afferents from its rostral nervous structures and sends afferents to pre-sympathetic and pre-parasympathetic neurons (See list of abbreviations).

The posterior hypothalamus at sites dorsal and medial to the fornix, the hypothalamic defense area (HDA) and the dorsal periaqueductal gray (PAG) zone are associated with the "defense reaction". The dorsolateral portion of the posterior hypothalamus, the hypothalamic vigilance area (HVA) and the ventrolateral PAG zone are part of the neurocircuit that mediates the "vigilance reaction". This circuit underlies affective responses to stressful stimuli and plays a fundamental role in integrating the effects of environmental events on cardiovascular regulation [62]. The paraventricular nucleus in the hypothalamus (PVN) is sympatho-excitatory and it is tonically activated by inputs that, in turn, are activated by increases in the level of circulating angiotensin II, chronic stress or anxiety, or peripheral receptors which may be tonically activated under certain conditions [63]. It is also one of the major direct projections to the NTS, as reported by Dampney [64]. It has even been suggested that the medial prefrontal cortex (mPFC) receives a variety of sensory information, including visceral signals, and helps the organism in selecting appropriate behavioral and autonomic responses for those stimuli and the emotional requirements of the situation [65-67]. The ventral part of the mPFC projects to the amygdala, among other neuronal structures [67]. Figure 3 summarizes the neural links briefly described above.

### Requirements from a control theory viewpoint

After all of the above and considering a control theory perspective, the NTS in the medulla oblongata would act as a comparator if and only if,

a) Its lesion suppressed the cardiovascular regulation;

b) the negative feedback loop still responded normally to perturbations (such as mechanical or electrical) after cutting the rostral afferent fibers to the NTS;

c) perturbation of a RNS path to the NTS modified the set point without changing the pattern, say, the dynamics of the elicited response; and

d) cardiovascular responses to perturbations on neural structures within the negative feedback loop compensated much faster than perturbations on the NTS rostral neural structures.

Several experimental data support each of the hypotheses above, such as,

a) the acute effects of central disruption of the baroreflexes were first studied in the rat by Doba and Reis, in 1973 [68] Bilateral electrolytic lesions of the NTS abolished the baroreflexes and produced fulminating hypertension due to a sympathetically mediated increase in total peripheral resistance. This was followed by cardiac failure, pulmonary edema and death within hours. Besides, the destruction of adrenergic terminals in the NTS with 6-OHDA (6-hydroxydopamine, substance used to kill dopaminergic and noradrenergic neurons) produces a permanent lability of blood pressure according to Talman *et al *[69]. These experiments show the relevant role of the NTS and that the mentioned secondary feedback loop is not relevant for the regulatory action. On the other hand,

b) the cardiovascular reflexes do not significantly change after decerebration [70]. Besides,

c) if during the drift in blood pressure elicited by electrical stimulation of a RNS (after the transient), a neural structure of the negative feedback loop is perturbed, the pattern of pressure response (amplitude and time to stabilization) is similar to that before stimulation. Covian, in 1966 [71], found in normotensive anesthetized rats, that the baroreceptor reflex is blocked due to the simultaneous stimulation of the septal area and the negative feedback loop (by carotid occlusion). In that paper can also be seen that after a few minutes of the septal stimulus withdrawal, the pattern of pressure response to loop stimulation (amplitude and time to stabilization) is similar to that before septal stimulation. In a more recent work from the same laboratory, Scopinho *et al *[72] found in normotensive conscious rats, that acute inhibition of lateral septal area by cobalt chloride (CoCl_2_) increases the gain of both brady- and tachycardiac responses to respectively mean arterial pressure increase or decrease for about tens of minutes. They proposed that the LSA exerts a tonic inhibitory role in the baroreflex modulation, affecting both the sympathetic and the parasympathetic components of the reflex. Besides, similar patterns of pressure response before and after 10 minutes of the injection of CoCl_2 _can be observed. This is to be expected if the lateral septal area affects the sympathetic and the parasympathetic components of the reflex. Similar baroreflex inhibition was reported in other areas connected with the lateral septal area [73], such as the hypothalamus [74], dorsolateral periaqueductal gray matter [75,76], medial prefrontal cortex [77,78], and the bed nucleus of the stria terminalis [79]. Finally,

d) when neural structures to the NTS are perturbed by electrical stimulation, the pressure response is slowly compensated for (it returns to the original value within tenths of a minute). This has been observed during stimulation of the lateral hypothalamus, the lateral and medial septum and the amygdala [80-83]. In contrast, if the perturbation is done inside the negative feedback loop, the pressure is compensated for much faster, returning to the original value within a few minutes [70]. These experiments showed that the rostral projections to the NTS do not belong to the feedback loop.

The conclusion is that, since all four proposed conditions are satisfied, the NTS does act as a comparator.

A few complementary considerations with good back up are pertinent. In adult animals, rostral neural structures to the NTS modulate the feedback loop responses [84,85]. Descending inputs from the hypothalamus and other supramedullary regions are activated as part of the response to an alerting or stressful stimulus; this results in modulation of the baroreceptor reflex, as mentioned by Spyer, in 1992 [5]. The lateral hypothalamus modulates cardiovascular variables in different behavioral situations as well as the responses to electrical stimulation on structures of the feedback loop [86,87]. Activation of the PVN causes inhibition of the baroreceptor reflex, as occurs in conditions where sympathetic activity is chronically increased, such as heart failure. It is interesting to note that the NTS mediates that inhibitory effect on the baroreceptor reflex [88,89]. Let us recall that responses from the baro and chemoreceptor blood pressure system can be elicited either by mechanical, say aortic or carotid compression, the former being illustrated by the old Chauveau-Marey maneuver [12,13], or electrical at the level of Hering's nerve or higher up in the different neural pathways. As examples, the lateral septum also modulates some cardiovascular responses to feedback loop perturbation, such as the bilateral carotid occlusion or the electrical stimulation on the ventrolateral reticular formation [71]. Moreover, the amygdala plays a special role in the regulation of the cardiovascular system during specific behavioral stress [56]. The cerebellum is another structure that contributes to the neural regulation of blood pressure [56,90-93]. The fastigial nucleus does not affect the cardiovascular variables in resting condition, but it plays a modulation role during exercise [94]. Moreover, secretions from the adrenal medulla have profound cardiovascular influences. With regard to sympathetic neurons, there are descending pathways to the pre-ganglionic neurons of the adrenal medulla, which stem at hypothalamic, midbrain, pontine and medullary cell groups [95].

## Discussion and Conclusions

Based on the fact that denervation of all the cardiovascular receptors (baro, chemo and cardiopulmonary) provoke sustained hypertension, we conclude that mean long-term blood pressure is regulated by the nervous system. We analyzed the cardiovascular neural circuit, particularly the open loop and the feedback loop closed by cardiovascular receptors. The NTS is the only structure that receives information from its RNS and from cardiovascular receptors and projects to nuclei that regulate the circulatory variables. There is also a secondary feedback loop closed by the LRN, but without showing salient importance in the long-term regulation, as remarked above in Section 4. When the NTS is injured, MAP cannot stabilize. On these bases and from a control theory point of view, we showed that this nucleus has the emergent property of a comparator and its afferents from the RNS provide the set point, which determines mean arterial pressure. Thus, the baroreflex would stabilize the instantaneous pressure value to the prevailing carotid pressure (MAP). In this way, the long-term control of arterial pressure occurs independently of arterial baroreceptor input. Such result is in agreement with Osborn [26].

Hypertension could be looked at as a set point shift due to the influence from higher level pathways, say, increased pressure because of a tumor, or small edematous areas originated in inflammation processes which, in turn, might be linked to autoimmunological reactions. Other causes, which might be termed as behavioral, are amenable in this context, as for example, the lateral septal area has been reported to modulate autonomic responses to stress and emotional situations [96]. Since baroreflex parasympathetic component is suppressed during stress [97], this area could be modulating the baroreflex parasympathetic component during defensive stress situations leading to an increase in blood pressure.

Mean arterial pressure (MAP) is regulated by two neural mechanisms: first, a negative feedback loop where the RNS to the NTS would function as the set point, and second, an open loop where several brainstem nuclei of the closed loop (with fibers to the sympathetic and parasympathetic systems) receive feedforward or open loop (FF, see note at the end of the paragraph) projections from the same RNS to the NTS. They have at least two functions: To determine the MAP feedback loop set-point modulating some neural loop structures. Since the cardiovascular feedback is too slow, the RNS to the NTS play a functional role not only under steady-state conditions but it may also vary according to the particular situation (say, different behaviors and pathologies). In hypertension, for example, stress might change some RNS inputs to the NTS, resulting in a set point modification. In short: In our view, the collected review including results from our own experimental data, gives enough support to the NTS as a neural comparator in blood pressure regulation.

In control theory, there are three basic mechanisms of regulation: buffering, feedforward and feedback. In each case, the effect of disturbances on the essential variables is reduced, either by a passive buffer, or by an active regulator in the two latter.

## Competing interests

The authors declare that they have no competing interests.

## Authors' contributions

This paper is the result of experiments performed by MEV years ago in the USA and experiments carried out by BSZ and ETS in Buenos Aires. BSZ propose the way to prove that the NTS functions as a comparator in MAP regulation. All authors participated in the study design, drafted the manuscript, and read and approved the final manuscript.

## List of Abbreviations or Acronyms

ANS: Autonomic Neural System; Ba: Baroreceptors; BP: Blood Pressure; CAC: Cardioacceleratoy Center; CP: Cardiopulmonary Receptors; Ch: Chemoreceptors; CIC: CardioInhibitory Center; CNS: Central Nervous System; CO: Cardiac Output; Comp: Comparator; CVLM: Caudal Ventro-Lateral Medulla; DMH: Dorso-Medial Hypothalamic nucleus; DVN: Dorsal Ventral Nucleus; HDA: Hypothalamic Defense Area; HVA: Hypothalamic Vigilance Area; IML: Inter-Medio-Lateral spinal column fibers; IVLM: Intermediate Ventro Lateral Medulla; LRN: Lateral Reticular Nucleus; mPFC: Medial Prefrontal Cortex; MAP: Mean Arterial Pressure; Med: Medulla Oblongata; NA: Nucleus Ambiguus; NGC: Nucleus Gigantocellularis; PAG: Dorsal Periaqueductal Gray matter; PR: Peripheral Resístanse; PVN: paraventricular nucleus of the hypothalamus; R: Neural Reference or Set Point (taken both terms as equivalent); RVLM: Rostral Ventro-Lateral Medulla; SV: Stroke Volume; VLM: Ventro-Lateral Medulla; VMC: Vasomotor Center; Trans: Transducers (postulated).
